# Association between hematopoietic recovery indicators and minimal residual disease after induction therapy in newly diagnosed pediatric acute myeloid leukemia

**DOI:** 10.3389/fmed.2026.1950889

**Published:** 2026-09-22

**Authors:** Wen-ting Pei, Chun-lei Liu, Xiao-ling Li

**Affiliations:** 1 Department of Pediatrics, Children’s Hospital Affiliated to Shandong University (Jinan Children’s Hospital), Jinan, Shandong Province, China; 2 Department of Information Technology, Children’s Hospital Affiliated to Shandong University (Jinan Children’s Hospital), Jinan, Shandong Province, China

**Keywords:** hematopoietic recovery, induction chemotherapy, minimal residual disease, pediatric acute myeloid leukemia, platelet recovery, predictive model, prognostic stratification

## Abstract

**Objective:**

This study aimed to investigate the relationship between peripheral blood and bone marrow hematopoietic recovery indicators and minimal residual disease (MRD) status following induction chemotherapy in pediatric patients with newly diagnosed acute myeloid leukemia (AML), and to evaluate the early predictive value of these indicators for MRD positivity.

**Methods:**

Clinical data from 62 pediatric patients with newly diagnosed AML who received standard induction chemotherapy between January 2015 to December 2024 were retrospectively reviewed. Patients were stratified into MRD-negative (MRD < 0.1%, *n* = 43) and MRD-positive group (MRD ≥ 0.1%, *n* = 19) groups based on post-induction MRD assessment. Hematopoietic recovery parameters, including nadir values and time to recovery of absolute neutrophil count (ANC) and platelet (PLT) count, as well as bone marrow cellularity and granulocytic/megakaryocytic recovery on day 28, were compared between groups. Spearman correlation analysis assessed associations between hematopoietic indicators and MRD levels. Multivariate logistic regression identified independent correlates of MRD positivity. Receiver operating characteristic (ROC) curves with DeLong's test compared predictive performance of individual and combined indicators. Least Absolute Shrinkage and Selection Operator (Lasso) regression with 10-fold cross-validation was used for variable selection. Internal validation was performed using 1000 bootstrap resamples.

**Results:**

The two groups were well-balanced in baseline characteristics, with no significant differences. Those in the MRD-positive group had significantly lower ANC and PLT nadirs, and prolonged time to ANC and PLT recovery. On day 28, a higher proportion of patients in the MRD-positive group exhibited hypocellular bone marrow, along with impaired granulocytic and megakaryocytic recovery. Spearman analysis showed that PLT recovery time was strongly positively correlated with MRD levels, while PLT nadir showed a strong negative correlation; both correlations were stronger than those observed for ANC-related indicators. Multivariate logistic regression confirmed that delayed PLT recovery and reduced PLT nadir were independent correlates of MRD positivity. The combination of PLT recovery time and PLT nadir achieved an area under the ROC curve (AUC) of 0.820 (95% CI: 0.724 - 0.916) for predicting MRD positivity, with a sensitivity of 84.37% and specificity of 78.21%, significantly outperforming individual indicators. Bootstrap validation confirmed model stability (AUC = 0.798, 95% CI: 0.712 - 0.884).

**Conclusion:**

Hematopoietic recovery indicators are closely associated with MRD status in pediatric AML following induction therapy. MRD-positive patients exhibit delayed and poor-quality hematopoietic recovery. Delayed PLT recovery and reduced PLT nadir are robust independent correlates of MRD positivity, and their combined use offers a simple, accessible tool for early MRD risk stratification and efficacy assessment, particularly in resource-limited settings.

## 1 Introduction

Acute myeloid leukemia (AML) is a highly aggressive clonal disorder arising from hematopoietic stem and progenitor cells, characterized by uncontrolled proliferation and differentiation arrest of myeloid blasts (1). Accounting for approximately 15% to 20% of childhood leukemias, AML exhibits greater biological heterogeneity, lower initial remission rates, and higher relapse risk compared with acute lymphoblastic leukemia (ALL), contributing to substantial variability in long-term outcomes (2). Epidemiological data indicate that the 5-year event-free survival (EFS) rate for pediatric AML ranges from 60% to 70%, whereas patients who relapse face a dismal 5-year survival rate below 30% (3). Therefore, early and accurate assessment of treatment response, timely identification of high-risk subgroups, and individualized treatment stratification are critical to improving long-term prognosis.

Induction chemotherapy remains the cornerstone of initial treatment for pediatric AML, with the primary goal of eradicating leukemic clones to achieve complete morphologic remission (CR), defined as bone marrow blasts < 5%, absence of peripheral blasts, and resolution of extramedullary disease. However, CR reflects leukemia clearance only to the threshold of conventional morphology, leaving subclinical residual disease undetected (4). Minimal residual disease (MRD), defined as residual leukemic cells below the morphologic detection limit (typically < 10^−2^ to 10^−3^), has emerged as one of the most robust independent prognostic factors in pediatric AML (5). With the widespread adoption of high-sensitivity techniques such as multiparameter flow cytometry (MFC) and real-time quantitative polymerase chain reaction (RQ-PCR), MRD has become a critical tool for guiding treatment intensification: patients with post-induction MRD ≥ 0.1% face significantly higher relapse risk and may benefit from intensified consolidation or hematopoietic stem cell transplantation (HSCT), whereas those with MRD <0.1% can often be managed with less aggressive regimens (6, 7).

Hematopoietic recovery, the process of bone marrow function restoration following chemotherapy-induced suppression, reflects both the degree of damage to normal hematopoietic stem and progenitor cells and the effectiveness of leukemic clone eradication (8). Pathophysiologically, complete clearance of leukemic cells allows normal hematopoiesis to resume unimpeded within a supportive bone marrow microenvironment, facilitating rapid and robust recovery (6). In contrast, persistent residual leukemia cells compete with normal hematopoietic progenitors for limited niches, cytokines (e.g., thrombopoietin, granulocyte colony-stimulating factor), and nutritional support, while also secreting inflammatory factors [e.g., tumor necrosis factor (TNF)-*α*, interleukin (IL)-6] that disrupt stromal function—collectively contributing to delayed or impaired hematopoietic recovery (9). Thus, indicators of hematopoietic recovery (e.g., neutrophil and platelet recovery kinetics, bone marrow cellularity) hold theoretical promise as surrogate markers of MRD status, particularly in settings where high-precision MRD testing is not readily available.

Despite this rationale, existing evidence on the association between hematopoietic recovery and MRD in pediatric AML has notable limitations. First, most studies have focused on adult populations, with limited data in children, whose hematopoietic reserves, chemotherapy tolerance, and disease biology differ substantially (6, 10). Second, available pediatric studies are often limited by small sample sizes and single-center designs, with a narrow focus on single parameters (e.g., neutrophil recovery time) rather than a comprehensive evaluation of multi-dimensional hematopoietic indicators. Third, the predictive value of different hematopoietic parameters remains insufficiently validated, and no practical combined predictive tool has been established for routine clinical use. Moreover, MRD detection requires specialized equipment, technical expertise, and high costs, with long turnaround times, limiting its accessibility in many primary care and resource-limited settings (10). This gap leaves many pediatric AML patients without timely response assessment, hindering optimal treatment decisions.

To address these gaps, the present study systematically evaluated multi-dimensional hematopoietic recovery indicators, including recovery kinetics, nadir values, and bone marrow morphology, in a cohort of children with newly diagnosed AML. The objectives were to: (1) compare hematopoietic recovery patterns between MRD-positive and MRD-negative patients; (2) identify independent hematopoietic correlates of MRD positivity; (3) evaluate the predictive performance of individual and combined indicators for MRD; and (4) propose a simple, accessible tool for early MRD risk stratification, particularly for resource-limited settings. Recently, genomic and molecular biomarkers have been increasingly incorporated into AML risk stratification. For instance, Prajapati et al. demonstrated the prognostic relevance of molecular markers in guiding post-remission therapy. While these molecular tools offer high precision, their accessibility remains limited in many centers. In this context, platelet recovery parameters could serve as a cost-effective, real-time functional readout of bone marrow health, complementing molecular and MRD assessments in an integrated, multi-layered prognostic model (11).

## 2 Materials and methods

### 2.1 Study population

This retrospective study included pediatric patients (aged 0–18 years) with newly diagnosed AML who received standard induction chemotherapy at the Department of Pediatric Hematology between January 2015 to December 2024.

Inclusion criteria were: (1) diagnosis of AML confirmed by bone marrow morphology, immunophenotyping, cytogenetics, and molecular biology (including fusion genes and mutations) according to the *Guidelines for the Diagnosis and Treatment of Pediatric Acute Myeloid Leukemia* (1); (2) receipt of standard induction chemotherapy (DA, IA, or MA regimen), with dosage and course adjusted for age, weight, and risk stratification. The treatment protocol was determined based on the National Comprehensive Cancer Network (NCCN) Guidelines for Pediatric AML and risk stratification. Risk stratification was performed using the European LeukemiaNet (ELN) criteria, categorizing patients into low-risk (e.g., t (8; 21), inv (16)), intermediate-risk (e.g., normal karyotype, NPM1 mutation without FLT3-ITD), and high-risk groups (e.g., KMT2A rearrangement, monosomy 5/7, FLT3-ITD high allelic ratio). (3) completion of bone marrow morphology assessment and MRD testing at 28 ± 2 days after induction; (4) availability of complete clinical and laboratory data, including baseline characteristics, treatment details, dynamic hematopoietic monitoring, MRD results, and follow-up; (5) follow-up duration of at least 6 months.

Exclusion criteria were: (1) acute promyelocytic leukemia (APL, FAB M3 subtype) due to distinct treatment strategies (all-trans retinoic acid plus arsenic trioxide) and prognostic profiles (12); (2) secondary leukemia (e.g., transformed from myelodysplastic syndrome or congenital hematological disorders), congenital bone marrow failure syndrome, or congenital immunodeficiency; (3) early death (<28 days post-chemotherapy) or treatment abandonment due to severe infection, fatal bleeding, or multi-organ failure; (4) receipt of interventions affecting hematopoietic reconstruction (e.g., HSCT, immunotherapy, targeted therapy) during induction; (5) inability to accurately assess hematopoietic recovery due to severe infection or frequent blood transfusions. Subgroup analyses stratified by age (<3 years vs. ≥3 years) were performed because children under 3 years of age exhibit distinct AML biology, higher frequency of KMT2A rearrangements, and different chemotherapy tolerance compared to older children, as previously reported (13).

### 2.2 Treatment regimens

All patients received standardized induction chemotherapy: DA: daunorubicin 40 mg/(m^2^·d) on days 1-3; cytarabine (Ara-C) 100 mg/(m^2^·d) on days 1-7; IA: idarubicin 12 mg/(m^2^·d) on days 1-3; Ara-C 100 mg/(m^2^·d) on days 1-7; MA: mitoxantrone 10 mg/(m^2^·d) on days 1-3; Ara-C 100 mg/(m^2^·d) on days 1-7.

Supportive care was standardized: (1) infection prevention: prophylactic cephalosporins or carbapenems; targeted antimicrobial therapy based on pathogen identification; (2) organ protection: proton pump inhibitors, hepatoprotective agents, and antiemetics; (3) hematopoietic support: single-donor platelet transfusion when platelet count <20 × 10⁹/L or with bleeding symptoms; packed red blood cell transfusion when hemoglobin < 0 g/L. Response to induction was categorized as: complete remission (CR, blasts <5% with ANC >1.0 × 10⁹/L and PLT >100 × 10⁹/L), complete remission with incomplete hematologic recovery (CRi, blasts <5% with ANC ≤1.0 × 10⁹/L or PLT ≤100 × 10⁹/L), or non-remission (blasts ≥5%) according to ELN criteria (14).

### 2.3 Data collection and definition

Clinical data were extracted from the electronic medical record and laboratory information systems, with double-entry and cross-validation to ensure accuracy.

#### 2.3.1 Baseline data

Demographic characteristics (age, sex), FAB classification (excluding M3), cytogenetic/molecular risk stratification (low, intermediate, high per guidelines), and baseline laboratory parameters [white blood cell (WBC) count, hemoglobin, platelet count, liver and kidney function, coagulation profile] were collected.

#### 2.3.2 Hematopoietic reconstruction indicators

Peripheral blood indicators: Daily complete blood counts were monitored. Nadir ANC and platelet count: lowest values recorded after induction. ANC and platelet recovery times: interval from chemotherapy completion to the first of two consecutive days with ANC ≥ 1.0 × 10^9^/L or platelet count ≥100 × 10^9^/L, as per European LeukemiaNet (ELN) 2017 recommendations (14).

Bone marrow indicators: Bone marrow cellularity was classified as markedly hypercellular, hypercellular, normocellular, hypocellular, or markedly hypocellular (hypocellular + markedly hypocellular defined as hypocellular marrow) according to standard hematopathology criteria (15). Good granulocytic recovery was defined as granulocytic lineage proportion ≥30% with blasts < 5%; good megakaryocytic recovery as ≥7 megakaryocytes per smear (1.5 cm × 3.0 cm) with no morphologic abnormalities (e.g., multinucleation, micromegakaryocytes), based on established criteria for pediatric bone marrow recovery (16).

#### 2.3.3 MRD detection

MRD was assessed by MFC using an 8-color antibody panel (CD45/CD34/CD13/CD33/CD117/HLA-DR/CD7/CD19) on a BD FACSCanto II flow cytometer (BD Biosciences), following ELN consensus guidelines (14). The gating strategy identified blast populations using CD45 vs. side scatter, followed by analysis of aberrant antigen expression patterns (e.g., CD34 + CD117 + HLA-DR– or cross-lineage CD7 expression) (17). For patients with specific molecular markers (AML1-ETO, CBFβ-MYH11, NPM1 mutations), RQ-PCR was performed concurrently (primer sequences and cycling conditions in Supplementary Table S1). MRD < 0.1% was defined as negative; MFC results were used as the gold standard in cases of discordance (4).

### 2.4 Ethical statement

This study was conducted in accordance with good clinical practice guidelines and the Declaration of Helsinki. The study protocol was approved by the local ethics committee, which waived the requirement for obtaining informed consent due to the retrospective nature of the study.

### 2.5 Statistical methods

Statistical analyses were performed using SPSS 26.0 and R 4.3.1 (rms, pROC, boot packages). Normality was assessed using the Shapiro–Wilk test. Continuous data are presented as mean ± standard deviation or median (interquartile range) and compared using independent t-tests or Mann–Whitney U tests. Categorical data are presented as frequencies (percentages) and compared using *χ*^2^ or Fisher's exact tests. Spearman rank correlation was used to assess associations between hematopoietic indicators and MRD levels.

Lasso regression with 10-fold cross-validation was used for variable selection (*λ* min = 0.086). Multivariate logistic regression identified independent correlates of MRD positivity, including remission status (CR vs. CRi vs. non-remission) as a covariate. ROC curves with DeLong's test compared predictive performance; AUC < 0.7 was considered low, 0.7–0.8 moderate, and ≥0.8 high. Model calibration was assessed using calibration curves and the Hosmer–Lemeshow test. Internal validation was performed with 1000 bootstrap resamples. Subgroup analyses stratified by age (<3 years vs. ≥3 years) and risk group (low/intermediate vs. high) evaluated model generalizability. A two-sided *P* < 0.05 was considered statistically significant.

## 3 Results

### 3.1 Baseline characteristics

A total of 62 patients were included [34 (54.83%) male, 28 (45.17%) female; aged 1–18 years (mean: 7.3 ± 3.6 years)]. FAB subtypes included M0 (0.00%), M1 (9.67%), M2 (46.77%), M4 (20.97%), M5 (22.59%), M6 (0%), and M7 (0%). Risk distribution was low (24.19%), intermediate (48.39%), and high (27.42%). Baseline median WBC was 28.5 (range 1.2–186.0) × 10^9^/L, hemoglobin 72 g/L (35 - 110), and platelet count 35 (5 - 120) × 10^9^/L.

43 patients (69.35%) were MRD-negative and 19 (30.65%) MRD-positive. Baseline characteristics were well balanced between groups, confirming comparability, as detailed in Table 1. Among the 62 patients, 46 (74.2%) achieved CR, 10 (16.1%) achieved CRi, and 6 (9.7%) were classified as non-remission after induction. In the MRD-negative group, 41 (95.3%) achieved CR and 2 (4.7%) achieved CRi. In the MRD-positive group, 5 (26.3%) achieved CR, 8 (42.1%) achieved CRi, and 6 (31.6%) were non-remission.

**Table 1:** Baseline characteristics of MRD-negative and MRD-positive groups.

Characteristic	MRD-negative (*n* = 43)	MRD-positive (*n* = 19)	Test Statistic	*P*-value
Sex, n (%)			*χ*^2^ = 0.012	0.913
Male	23 (53.49)	11 (57.89)		
Female	20 (46.51)	8 (42.11)		
				
Age group, n (%)			χ^2^ = 0.357	0.837
<3y	7 (16.28)	3 (15.79)		
3～10y	24 (55.81)	12 (63.16)		
>10y	12 (27.91)	4 (21.05)		
FAB subtype, n (%)			χ^2^ = 1.208	0.973
M0	0 (0)	0 (0)		
M1	4 (9.30)	2 (10.53)		
M2	20 (46.51)	9 (47.37)		
M4	9 (20.93)	4 (21.05)		
M5	10 (23.26)	4 (21.05)		
M6	0 (0)	0 (0)		
M7	0 (0)	0 (0)		
Risk group, n (%)			χ^2^ = 0.872	0.647
Low	10 (23.26)	5 (26.32)		
Intermediate	21 (48.84)	9 (47.36)		
High	12 (27.90)	5 (26.32)		
Initial WBC, ×10^9^/L, M (Q1, Q3)	27.8 (8.5, 65.2)	30.2 (10.3, 72.5)	Z = −0.428	0.668
Initial Hb, g/L, M (Q1, Q3)	73 (55, 90)	70 (52, 88)	Z = −0.536	0.592
Initial PLT, ×10^9^/L, M (Q1, Q3)	36 (18, 65)	33 (15, 60)	Z = −0.385	0.700
Remission status, n (%)			χ^2^ = 16.245	<0.001
CR	41 (95.3)	5 (26.3)		
Cri	2 (4.7)	8 (42.1)		
Non-remission	0 (0)	6 (316)		

WBC, white blood cell; Hb, hemoglobin; PLT, platelet; CR, complete remission; CRi, complete remission with incomplete hematologic recovery; χ^2^, chi-square; Z, Mann–Whitney U.

### 3.2 Comparison of hematopoietic recovery indicators

#### 3.2.1 Peripheral blood indicators

Table 2 showed that the MRD-positive group had significantly lower ANC and platelet nadirs and longer recovery times, compared with the MRD-negative group. The most pronounced difference was observed for platelet recovery time (Z = −5.623, *P* < 0.05).

**Table 2:** Comparison of peripheral blood hematopoietic recovery indicators [M (Q1, Q3)].

Indicator	MRD-negative (*n* = 43)	MRD-positive (*n* = 19)	Z-value	*P*-value
ANC nadir, ×10^9^/L	0.12 (0.06, 0.20)	0.08 (0.03, 0.15)	−2.876	<0.05
ANC recovery time, days	18 (15, 22)	25 (20, 30)	−4.982	<0.05
PLT nadir, ×10^9^/L	12 (8, 18)	8 (5, 12)	−3.965	<0.05
PLT recovery time, days	22 (19, 26)	31 (27, 35)	−5.623	<0.05

ANC, absolute neutrophil count; PLT, platelet.

#### 3.2.2 Bone marrow indicators

At day 28 (± 2 days) post-induction, the MRD-positive group had a higher proportion of hypocellular marrow (64.71% vs. 28.21%), lower rates of good granulocytic recovery (44.12% vs. 76.92%), and lower rates of good megakaryocytic recovery (38.24% vs. 71.79%) (all *P* < 0.05) (Table 3).

**Table 3:** Comparison of bone marrow hematopoietic recovery indicators [n (%)].

Indicator	MRD-negative (*n* = 43)	MRD-positive (*n* = 19)	χ^2^-value	*P*-value
Bone marrow cellularity			16.892	<0.05
Hypocellular	12 (27.91)	13 (68.42)		
Non-hypocellular	31 (72.09)	6 (31.58)		
Granulocytic recovery			13.568	<0.05
Good	33 (76.74)	8 (42.11)		
Poor	10 (23.26)	11 (57.89)		
Megakaryocytic recovery			14.235	<0.05
Good	31 (72.09)	7 (36.84)		
Poor	12 (27.91)	12 (63.16)		

### 3.3 Correlation between hematopoietic indicators and MRD levels

Platelet recovery time (r = 0.840) and platelet nadir (r = −0.754) showed stronger correlations with MRD levels than ANC recovery time (r = 0.521) and ANC nadir (r = −0.490) (Figure 1).

**Figure 1:**
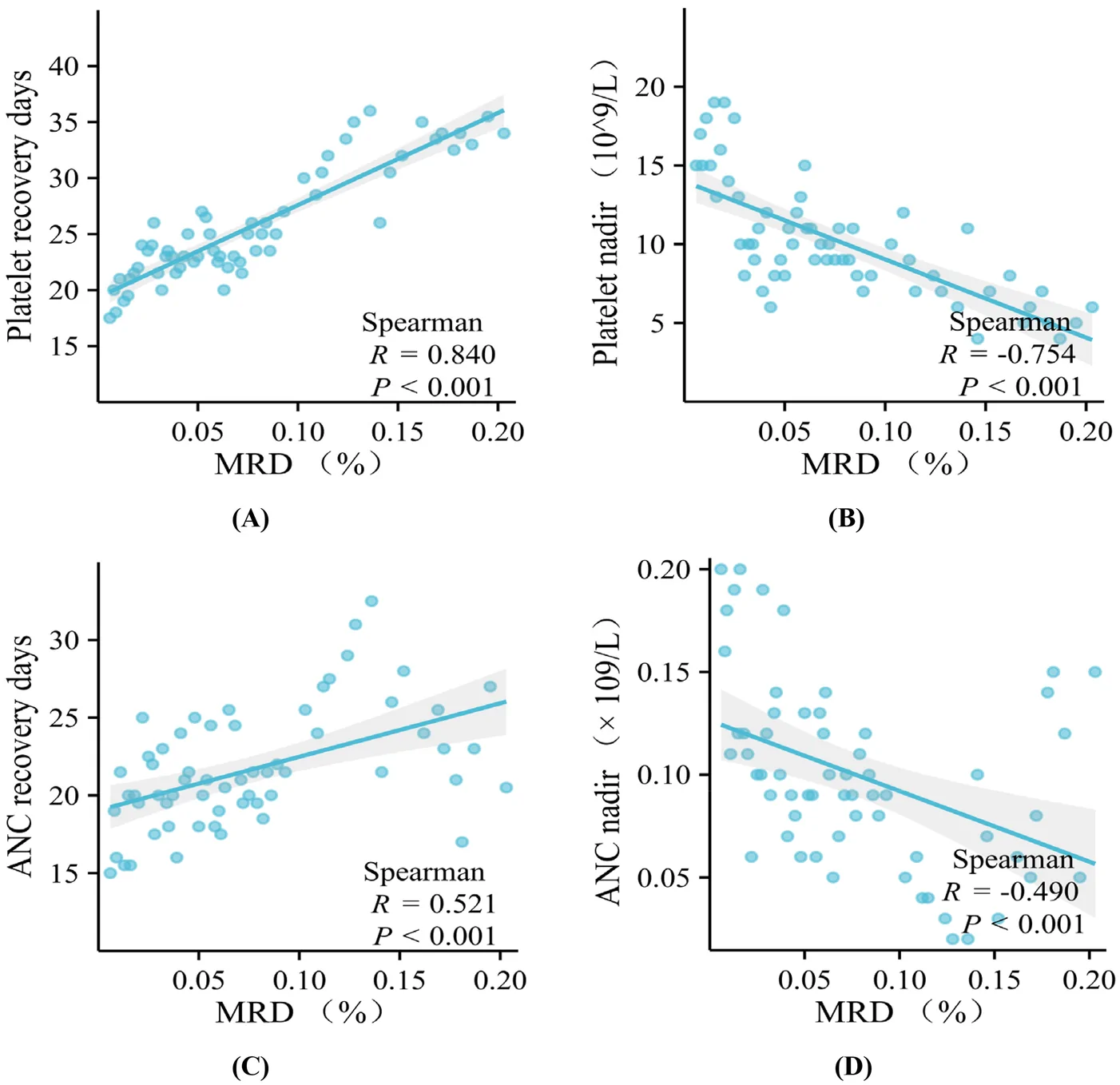
Scatter plots showing correlations between hematopoietic indicators and MRD levels. **(A)** Platelet recovery time vs. MRD; **(B)** platelet nadir vs. MRD; **(C)** ANC recovery time vs. MRD; **(D)** ANC nadir vs. MRD. Blue lines represent trend lines; r = Spearman correlation coefficient.

### 3.4 Independent correlates of MRD positivity

Multivariate logistic regression, adjusting for remission status (CR/CRi/non-remission), identified delayed platelet recovery (OR = 3.872, 95% CI: 1.765 - 8.483) and reduced platelet nadir (OR = 2.945, 95% CI: 1.328 - 6.527) as independent correlates of MRD positivity (Table 4). Remission status was not independently associated with MRD positivity after adjusting for platelet recovery parameters (*P* = 0.214).

**Table 4:** Multivariate logistic regression analysis of MRD positivity.

Variable	*β*	SE	OR	95% CI	*P*-value
ANC recovery time (days)	0.032	0.021	1.033	0.991–1.076	0.128
PLT recovery time (days)	0.136	0.042	3.872	1.765–8.483	0.001
ANC nadir (×10^9^/L)	−0.875	0.562	0.416	0.145–1.192	0.102
PLT nadir (×10^9^/L)	−0.982	0.376	2.945	1.328–6.527	0.008
Hypocellular marrow (yes = 1)	0.653	0.412	1.921	0.897–4.118	0.098
Remission status (CR as reference)					
CRi vs. CR	0.412	0.324	1.510	0.801–2.846	0.214
Non-remission vs. CR	0.561	0.389	1.752	0.816–3.762	0.151

β, regression coefficient; SE, standard error; OR, odds ratio; CI, confidence interval; CR, complete remission; CRi, complete remission with incomplete hematologic recovery.

### 3.5 Predictive performance of hematopoietic indicators

The combination of platelet recovery time and platelet nadir achieved the highest AUC (0.820, 95% CI: 0.724 - 0.916), significantly outperforming individual indicators (DeLong's test, *P* < 0.05) (Figure 2). Calibration curves showed good agreement between predicted and observed MRD positivity (Hosmer–Lemeshow test, *χ*^2^ = 8.216, *P* = 0.412). Bootstrap validation confirmed stable performance (AUC = 0.798, 95% CI: 0.712 - 0.884). Subgroup analyses demonstrated consistent predictive performance across age groups (AUC = 0.800 for <3 years vs. 0.815 for ≥3 years, *P* for interaction = 0.723) and risk groups (AUC = 0.796 for low/intermediate risk vs. 0.829 for high risk, *P* for interaction = 0.615) (Figure 3).

**Figure 2:**
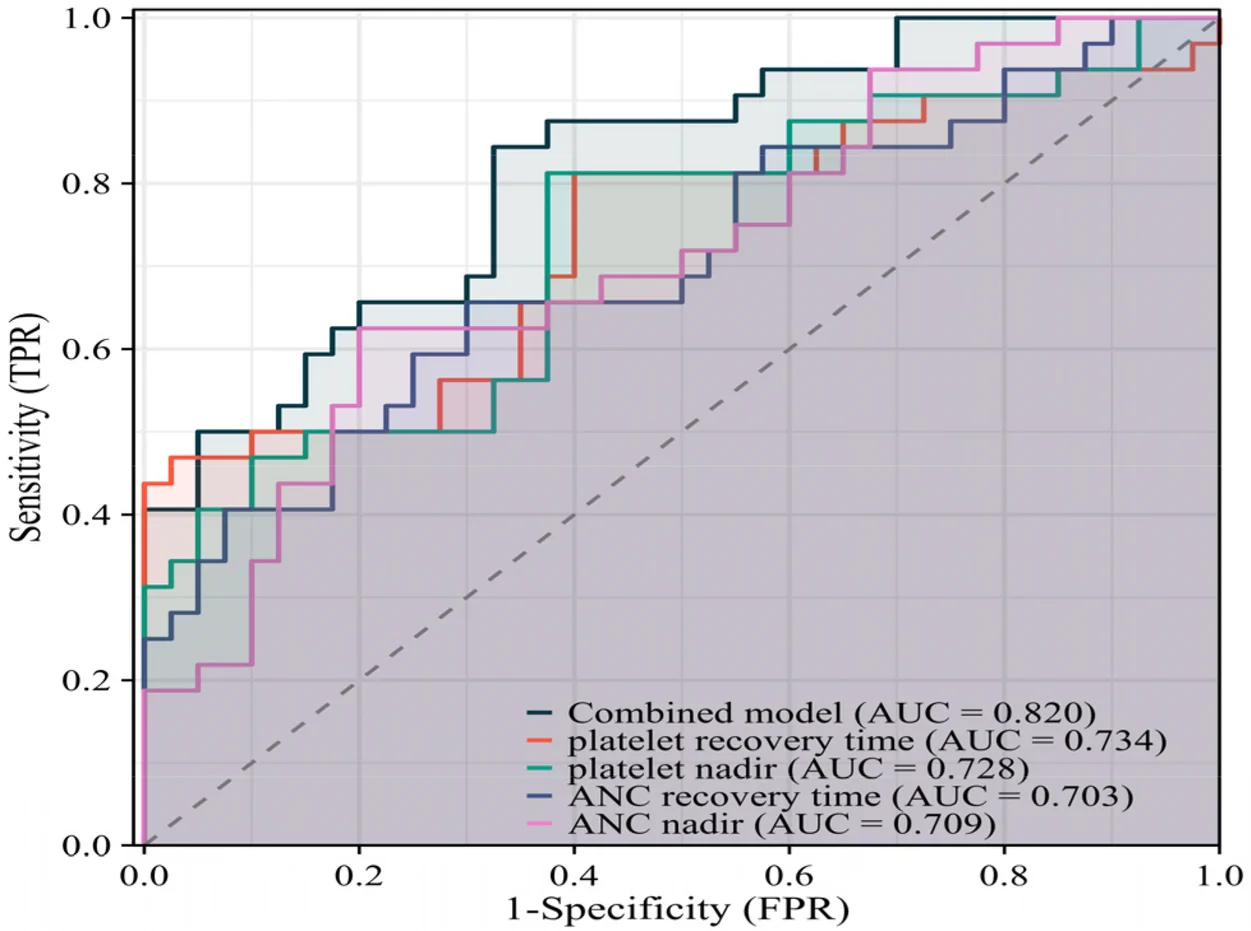
The predictive efficacy for MRD positivity between the combined model and single indicators.

**Figure 3:**
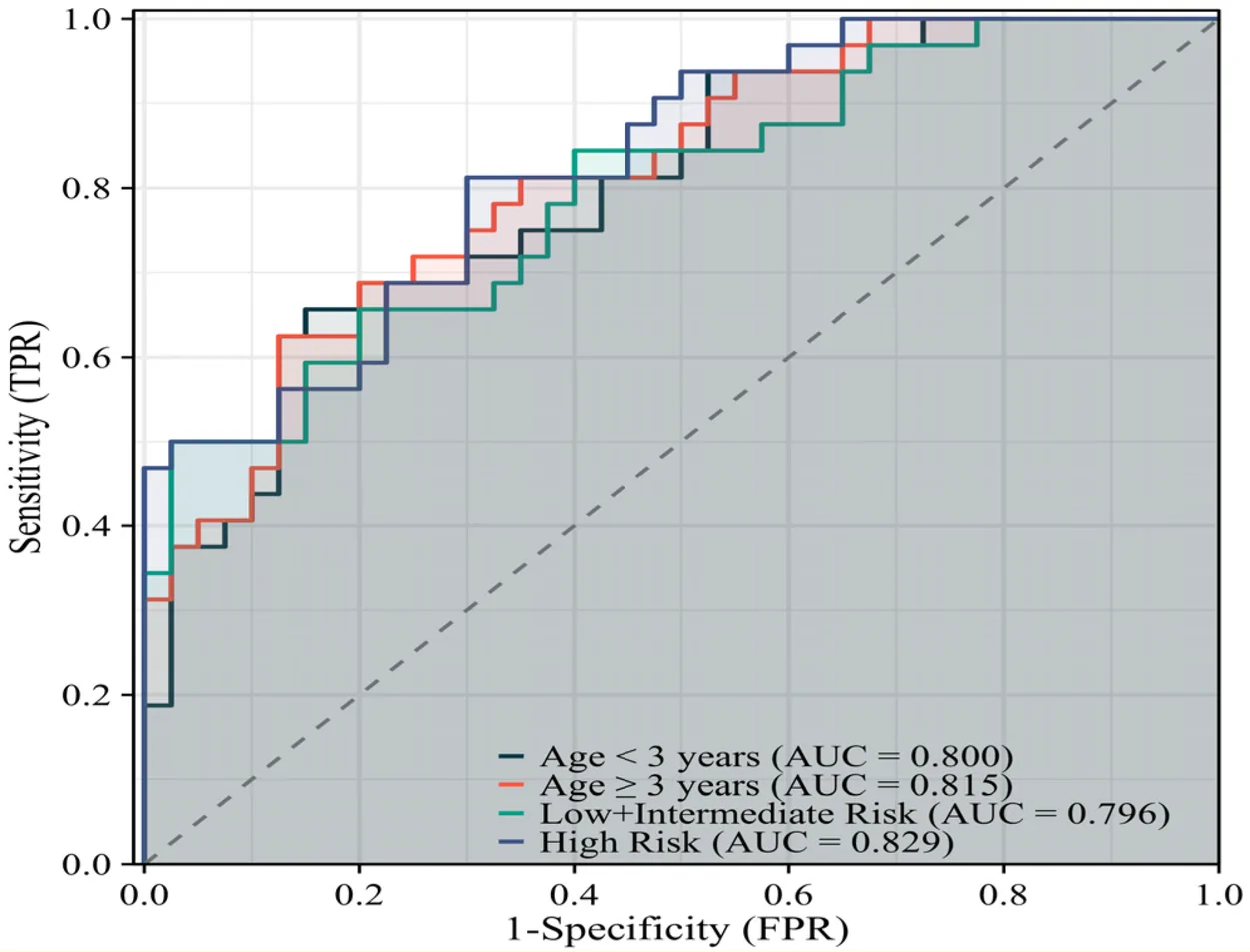
The predictive efficacy of subgroup analyses (age, risk) for MRD positivity.

## 4 Discussion

This study systematically evaluated multi-dimensional hematopoietic recovery indicators in a cohort of children with newly diagnosed AML and demonstrated three principal findings. First, MRD-positive patients exhibited a distinct pattern of impaired hematopoietic recovery, characterized by lower ANC and platelet nadirs, prolonged recovery times, and inferior bone marrow recovery at day 28 (± 2 days). Second, platelet-related indicators, particularly recovery time and nadir, showed stronger associations with MRD status than ANC-related parameters. Third, the combination of platelet recovery time and platelet nadir provided robust predictive performance for MRD positivity, offering a simple and accessible clinical tool, especially for resource-limited settings.

The superior predictive value of platelet-related indicators may be explained by the distinct biological features of megakaryocytic hematopoiesis. Megakaryocytes have a longer differentiation cycle (approximately 2-4 weeks) and are more sensitive to disruption of the bone marrow microenvironment compared with neutrophils (18). Residual leukemic cells compete with megakaryocyte progenitors for thrombopoietin (TPO) and stromal support, thereby inhibiting megakaryocyte proliferation and maturation (17). Furthermore, residual leukemic cells reshape the bone marrow niche by inducing a pro-inflammatory microenvironment, characterized by elevated TNF-α, IL-6, and TGF-β, which directly impair megakaryocyte differentiation and reduce TPO receptor (c-MPL) expression on hematopoietic progenitors (19). These niche alterations further suppress thrombopoiesis beyond the direct competition for TPO. In contrast, neutrophil recovery is typically faster (1-2 weeks); although G-CSF can accelerate neutrophil recovery, its routine use during AML induction is not generally recommended due to concerns about stimulating residual leukemic cells and lack of clear survival benefit (20, 21). Therefore, neutrophil recovery may be less confounded by exogenous growth factors in most clinical settings, yet its faster kinetics still make it a less sensitive indicator of low-level residual disease compared to platelet recovery. These mechanistic differences likely support the hypothesis why platelet recovery indicators serve as more reliable surrogates for MRD in pediatric AML, consistent with recent findings in adults (6), while extending this observation to children with distinct hematopoietic physiology.

The findings address an important clinical gap in pediatric AML management, particularly in resource-limited settings where MRD testing is not widely available. In many primary centers, MFC- or RQ-PCR-based MRD detection is limited by high costs, technical requirements, and long turnaround times. The proposed combined platelet-based tool (AUC = 0.820) provides a pragmatic alternative: monitoring platelet recovery time (> 30 days) and nadir (< 8 × 10^9^/L) can help identify high-risk patients who warrant confirmatory MRD testing. We propose a practical workflow: For patients with platelet recovery time >30 days or platelet nadir <8 × 10⁹/L, early MRD testing should be prioritized. If MRD is negative, supportive care with TPO receptor agonists may be considered to expedite hematopoietic recovery without treatment escalation. If MRD is positive, these patients should be prioritized for early HSCT evaluation or intensified consolidation. This strategy ensures that platelet recovery parameters serve as a triage tool that complements, rather than replaces, standard MRD assessment. For patients with delayed platelet recovery but negative MRD, supportive care, such as TPO administration, may be optimized to accelerate hematopoietic recovery without unnecessary treatment escalation. Conversely, MRD-positive patients with poor platelet recovery may be prioritized for early HSCT evaluation or intensified consolidation to reduce relapse risk (22, 23). Furthermore, hematopoietic recovery indicators are available within 2-4 weeks after induction, enabling earlier risk stratification compared with conventional day 28 morphology or MRD results, facilitating timely treatment adjustments that are critical for improving outcomes in high-risk pediatric AML (5).

This study advances prior research in several ways. First, it takes a comprehensive approach by evaluating multiple dimensions of hematopoietic recovery, including peripheral blood kinetics and bone marrow morphology, rather than focusing on single parameters. Second, it proposes a practical combined predictive tool with demonstrated generalizability across age and risk subgroups, filling a gap in actionable surrogate markers for MRD in routine practice.

Our findings are consistent with Shahin et al. (6), who reported that incomplete hematologic recovery was associated with higher relapse risk in pediatric AML patients with negative MRD after induction. However, our study extends these observations by demonstrating that platelet-specific parameters outperform ANC-related indicators in predicting MRD positivity, and by providing a validated combined predictive model with defined clinical thresholds. Compared to adult studies that have emphasized ANC recovery as a prognostic marker (14), our results highlight the importance of age-specific markers, as pediatric hematopoietic progenitors may be more sensitive to microenvironmental disruption, making platelet recovery a more sensitive indicator in children.

Several clinical factors may influence platelet recovery kinetics and potentially confound the association with MRD. Infections are common after induction chemotherapy and can prolong bone marrow suppression through inflammatory cytokine release and direct bone marrow toxicity, thereby delaying platelet recovery independently of MRD status (24). Platelet transfusions, while necessary to prevent bleeding, may transiently affect platelet count measurements, although their impact on recovery kinetics is typically short-lived. Thrombopoietin administration, though not routinely used in our cohort, could accelerate platelet recovery and mask the inhibitory effects of residual leukemia. Bleeding complications may also increase platelet consumption, prolonging apparent recovery time. In our study, we applied strict exclusion criteria to minimize these confounders (e.g., excluding patients with severe infections or frequent transfusions). However, we acknowledge that residual confounding cannot be completely excluded, and future prospective studies should systematically collect data on these variables to adjust for their effects. Notably, our findings differ from some adult AML studies that have emphasized ANC recovery time as a prognostic indicator, underscoring the importance of age-specific prognostic markers. Pediatric hematopoietic stem cells possess greater proliferative capacity but may be more susceptible to microenvironmental disruption, making platelet recovery a more sensitive indicator of residual disease in children. This age-specific divergence highlights the need for pediatric-focused research rather than direct extrapolation of adult data. Although molecular biomarkers such as FLT3-ITD, NPM1 mutations, and KMT2A rearrangements provide critical prognostic information and guide targeted therapy, they do not directly reflect the functional status of the bone marrow microenvironment or real-time hematopoietic recovery. Platelet recovery parameters offer a complementary, dynamic assessment of marrow functional reserve that integrates the cumulative effects of disease burden, chemotherapy toxicity, and niche integrity. An integrated model combining molecular risk group, MRD status, and platelet recovery parameters could potentially refine prognostic stratification, especially in settings where MRD is not routinely available.

The choice of 3 years as the age cutoff for subgroup analysis was based on established pediatric AML biology: infants and young children under 3 years have a higher incidence of KMT2A rearrangements, distinct gene expression profiles, and different chemotherapy-related toxicity and outcomes compared to older children (13). This age threshold is commonly used in pediatric AML risk stratification and clinical trials, allowing for meaningful subgroup comparisons while maintaining sufficient sample sizes in each group.

Several limitations should be acknowledged. First, the retrospective, single-center design may introduce selection bias; multi-center prospective studies are needed to validate the generalizability of the prediction model. Second, molecular markers such as FLT3-ITD and NPM1 mutations were not integrated into the predictive model; future studies combining hematopoietic indicators with genetic data may further improve predictive accuracy. Integrating platelet recovery parameters with molecular risk stratification in a weighted prognostic score could enhance personalized treatment decisions and warrants investigation in future studies. Third, the potential impact of supportive care interventions (e.g., TPO administration) on hematopoietic recovery and MRD assessment was not evaluated and warrants investigation in future interventional studies.

## 5 Conclusion

Hematopoietic recovery indicators are closely associated with MRD status in children with newly diagnosed AML following induction chemotherapy. Delayed platelet recovery and reduced platelet nadir are robust independent correlates of MRD positivity, and their combined use provides a simple, accessible tool for early MRD risk stratification. This approach addresses a critical unmet need in resource-limited settings and facilitates timely treatment optimization, with the potential to improve outcomes for pediatric AML patients. These hematopoietic indicators should be viewed as complementary to, rather than a replacement for, standard MRD assessment, providing a practical triage tool that can prioritize patients for confirmatory MRD testing and guide early clinical decisions.

## Data Availability

The original contributions presented in the study are included in the article/Supplementary Material, further inquiries can be directed to the corresponding author.
