# Supplementary material for: Association between hematopoietic recovery indicators and minimal residual disease after induction therapy in newly diagnosed pediatric acute myeloid leukemia
Source: Front Med (Lausanne). 2026 Sep 22;13:1950889. doi: 10.3389/fmed.2026.1950889 (PMC13638423; doi:10.3389/fmed.2026.1950889)
Supplement: Supplementary file 1 [file Supplementaryfile1.doc]

**Supplementary Table S1:** Primer sequences and cycling conditions for RQ-PCR-based MRD detection

| **Molecular marker** | **Primer sequence (5’→3’)** | **Cycling conditions** | **Amplicon length (bp)** |
| --- | --- | --- | --- |
| AML1-ETO | Forward: TGTGCTGACCGAGGAGATG  Reverse: CAGAGCAGCCACGATGTC  Probe:  FAM-CTGCGGAAGATCGAGAAGCTG-TAMRA | 95℃ 10 min; 40 cycles (95℃ 15 s, 60℃ 60 s) | 128 |
| CBFβ-MYH11 | Forward: GCTGCTGCTGCTGCTGCT  Reverse: GCTGCTGCTGCTGCTGCTG  Probe: FAM-CTGCTGCTGCTGCTGCTGCTG-TAMRA | 95℃ 10 min; 40 cycles (95℃ 15 s, 62℃ 60 s) | 142 |
| NPM1 | Forward: GGTGGTGGTGGTGGTGGT  Reverse: CACCACCACCACCACCAC  Probe: FAM-ACCACCACCACCACCACCAC-TAMRA | 95℃ 10 min; 40 cycles (95℃ 15 s, 58℃ 60 s) | 116 |
